# Detection and differentiation of *Entamoeba histolytica* and *Entamoeba dispar* in clinical samples through PCR-denaturing gradient gel electrophoresis

**DOI:** 10.1590/1414-431X20175997

**Published:** 2017-04-03

**Authors:** P. López-López, M.C. Martínez-López, X.M. Boldo-León, Y. Hernández-Díaz, T.B. González-Castro, C.A. Tovilla-Zárate, J.P. Luna-Arias

**Affiliations:** 1 Family Medicine Unit 43, Social Security Mexican Institute, Cardenas, Tabasco, Mexico; 2 Health Sciences Academic Division, Juarez Autonomous University of Tabasco, Villahermosa, Tabasco, Mexico; 3 Multidisciplinary Academic Division at Jalpa de Mendez, Juarez Autonomous University of Tabasco, Jalpa de Mendez, Tabasco, Mexico; 4 Multidisciplinary Academic Division at Comalcalco, Juarez Autonomous University of Tabasco, Comalcalco, Tabasco, Mexico; 5 Center for Research and Advanced Studies, National Polytechnic Institute, Mexico City, Mexico

**Keywords:** Amebiasis, Neglected diseases, Diagnostic, PCR, DGGE, *adh112* gene

## Abstract

Amebiasis is one of the twenty major causes of disease in Mexico; however, the diagnosis is difficult due to limitations of conventional microscopy-based techniques. In this study, we analyzed stool samples using polymerase chain reaction-denaturing gradient gel electrophoresis (PCR-DGGE) to differentiate between *Entamoeba histolytica* (pathogenic) and *E. dispar* (non-pathogenic). The target for the PCR amplification was a small region (228 bp) of the *adh112* gene selected to increase the sensitivity of the test. The study involved 62 stool samples that were collected from individuals with complaints of gastrointestinal discomfort. Of the 62 samples, 10 (16.1%) were positive for *E. histolytica* while 52 (83.9%) were negative. No sample was positive for *E. dispar*. These results were validated by nested PCR-RFLP (restriction fragment length polymorphism) and suggest that PCR-DGGE is a promising tool to differentiate among *Entamoeba* infections, contributing to determine the specific treatment for patients infected with *E. histolytica,* and therefore, avoiding unnecessary treatment of patients infected with the non-pathogenic *E. dispar*.

## Introduction

One of the major health problems in developing countries is amebiasis. In 1997, the World Health Organization declared this disease as the third leading cause of death due to parasitic infections (1,2). Currently, amebiasis is still a serious public health problem because parasitic infections are commonly neglected, particularly in populations that lack hygienic measures and clean drinking water (1,3). In Mexico, amebiasis is one of the twenty major causes of disease; its incidence rate in 2000 was 1,353.43 per 100,000 (1,4,5).

Amebiasis is caused by the parasite *Entamoeba histolytica*, including both intestinal and extra-intestinal infections. This parasite can be present in sewage and contaminated water. According to its cell cycle, it can exist in two forms: trophozoites and cysts (6,7). There is a second species with identical morphological characteristics to those described for *E. histolytica* called *Entamoeba dispar;* however, the biochemical, immunological and genetic data indicate that *E. dispar* is non-pathogenic (8). The life cycle in both species is the same. The infection begins with the ingestion of cysts from water or food contaminated with fecal matter. In the small intestine occurs the excystation and the trophozoites emerge. The trophozoites colonize the large intestine and adhere to the colonic mucosa (6,9). Only the not encysted trophozoites of *E. histolytica* acquire invasiveness. By the action of proteases, hyaluronidases and mucopolysaccharidases *E. histolytica* erodes the mucosa producing ulcers and may even reach the submucosa. The adhesive interaction of the trophozoites with the surface of host cells is determinant for the invasion of human tissues, cytotoxic activity, and severity of the disease (10). Primary molecules involved in the intestinal invasion process of *E. histolytica* are the Gal/Gal NAc lectin and EhCPADH112 (124 kDa) complex (11,12). This complex is formed by the genes: i) *Ehcp112*, encoding a cysteine protease (50 kDa), and ii) *Ehadh112*, encoding an adhesin (75 kDa). Some studies have analyzed the molecular role of EhCPADH112 in *E. histolytica*, but it has not been identified in *E. dispar* (13).

The laboratory diagnosis of amebiasis is usually based on microscopy, immunological methods and polymerase chain reaction (PCR). The occurrence of non-pathogenic species (particularly *E. dispar*) causes a confusing scenario for a correct diagnosis of intestinal amebiasis and *E. histolytica* is often inaccurately reported or diagnosed (8,14–16). The denaturing gradient gel electrophoresis (DGGE) is a well-established tool for molecular microbiology; this method makes possible the electrophoretic separation of DNA fragments on the basis of differences in nucleotide composition rather than their size (17,18).

To address the need for a reliable diagnostic test of amebiasis caused by *E. histolytica* in human stools, we developed a polymerase chain reaction–denaturing gradient gel electrophoresis (PCR–DGGE), which differentiates *E. histolytica* from *E. dispar*, as it is highly specific and sensitive to these two species (more specific than other techniques such as ELISA). The PCR primers were based on a conserved portion of the *adh112* gene spanning a region with substitutions that allowed the differentiation between pathogen and non-pathogen species. To our knowledge, there are no studies using DGGE as a molecular identification technique to determine the prevalence of *E. histolytica* in Mexico.

## Material and Methods

### Sample details

The study involved 62 stool samples collected from patients who presented gastrointestinal complaints and attended the "Maximiliano Dorantes" Health Center. They were all examined for intestinal parasites using coproparasitoscopic studies (for multiple ova and parasites); we used the method of Faust with subsequent staining with Lugol solution in order to find cysts (19,20). We used *E. histolytica* DNA as a positive control, which was obtained from a monogenic culture of strain HM-1 IMSS donated by the Molecular Diagnostic Laboratory, Department of Experimental Pathology CINVESTAV. Likewise, DNA of *E. dispar* was also used as a positive control; this was donated by the Department of Experimental Medicine, Faculty of Medicine UNAM. The coproparasitoscopic tests were performed in triplicate.

### DNA extraction

The DNA of controls (*E. histolytica* and *E. dispar*) as well as the 62 studied samples were obtained directly from stools stored at –20°C, by means of *Entamoeba* cysts mechanical lysis, using zircon beads of 0.01 mm in diameter and then using the Wizard® Genomic DNA Purification Kit (Promega, USA) following the manufacturer's recommendations. As negative controls, we used human DNA extracted from whole blood of volunteer donors. We also utilized the Wizard® Genomic DNA Purification Kit to isolate DNA from white blood cells. The DNA samples were stored at –20°C until analyzed.

### Bioinformatic analysis for primer design

In order to differentiate the orthologous *adh112* gene of *E. dispar* from the adhesin of *E. histolytica*, a basic local alignment search tool (BLAST) was performed using the sequence reported by Garcia-Rivera et al. (21) obtained from GenBank database with accession number: AF127375 (*E. histolytica)*. Taking this as a target sequence, it was aligned using the BLAST tool in the genome database of all organisms to find highly conserved regions. *E. dispar* showed 93% sequence identity with the adhesin gene from *E. histolytica* (accession No.: AANV02000421).

**Figure 1:**
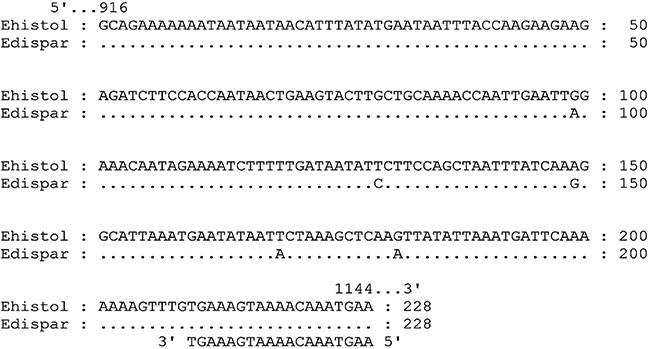
Alignment sequence between the *adh112* gene fragments from *E. histolytica* and *E. dispar*. Five differences between sequences are highlighted.

According to the high similarity between the two sequences, it was necessary to select a part of the sequence containing five differences for at least one nucleotide, because the DGGE is sensitive enough to detect differences of a single base between two sequences. The BioEdit (22) and GeneDoc (http://www.psc.edu/biomed/genedoc) softwares were used to interpret multiple alignments and manual adjustments. The alignment of *E. histolytica* and *E. dispar* sequences allowed the selection of a 228 bp-region containing five single base differences throughout the sequence (from base 916 to 1144) to be further amplified by PCR (Figure 1).

An *in silico* analysis to examine the complementarity within the sequence of each primer was performed using the Oligonalyzer program (http://www.idtdna.com/Home/Home.aspx). The designed primers also amplified another species of amoeba: *E. nuttalli*; however, its selected 228 bp-region did not contain the five single base differences observed in *E. histolytica* and *E. dispar*, so it was not included in our analysis. Moreover, *E. nuttalli is* non-pathogenic for humans and therefore, not important for our study (23). In order to achieve greater sensitivity in the detection by DGGE between *E. histolytica* and *E. dispar*, a GC-clamp was added to the forward primer: 5′-CGCCCGCCGCGCGGCCGCGGCCGGCCGGGGGCACGCGGCG-3′ (24), changing the total size of the fragment to be amplified from 228 to 268 bp.

### PCR amplification of *adh111* gene

We tested various quantities of DNA (25, 50, and 100 ng) for PCR amplification and decided to use 100 ng of DNA. For the first amplification, a reaction volume of 25 µL comprised: 4 µL of 10xPCR buffer (Invitrogen, USA), 3.2 µL of MgCl_2_ (50 mM; Invitrogen, USA), 1 µL of dNTP's mix (10 mM each; Invitrogen), 0.3 µL of each primer (40 µM), 0.2 µL (5 U/µL) of Accu Prime™ TaqDNA Polymerase High Fidelity (Invitrogen, USA), 2 µL (100 ng) of DNA and 14 µL of sterile deionized water. Finally, 2 drops of mineral oil were added. The amplification program of DNA started with 2 min of denaturation at 94°C, followed by 40 cycles of 60 s at 92°C of denaturation, primer annealing for 60 s at 47°C and extension for 90 s at 72°C. The final extension was at 72°C for 7 min. We used the primers: Fw 5′-GCA GAA AAA AAT AAT AAT AAC-3′ and Rv 5′-TTC ATT TGT TTT ACT TTC A-3′. After the first PCR, 5 µL of the amplified product were used for a second amplification under the same conditions mentioned above, using the primers: Fw 5′-CGC CCG CCG CGC GGC CGC GGC CGG CCG GGG GCA CGC GGC GGC AGA AAA AAA TAA TAA TAA C-3′ and Rv 5′-TTC ATT TGT TTT ACT TTC A-3′. Both amplifications were done in triplicate. PCR amplification products were verified on 1.6% (w/v) agarose gel using electrophoresis containing ethidium bromide.

### Denaturing gradient gel electrophoresis

The PCR products were subjected to DGGE with 10 and 30% linear denaturing gradients of urea and formamide in a 10% polyacrylamide gel (Promega, USA). DGGE was performed with a 10–30% denaturing gradient adding 210 µL of 10% ammonium persulfate and 10 µL of tetramethylethylenediamine (TEMED). The electrophoresis was pre-run in 1xTAE buffer (40 mM Tris, 20 mM acetic acid, 1 mM EDTA, pH 8.0) at constant 200 V during 15 min and at 60°C using the Bio-Rad (USA) D-Code TM Universal Mutation Detection System. The final conditions for electrophoresis gels were 5 h and 30 min at 130 V; the presence of PCR products was visualized by gel staining with 1 µg/mL of ethidium bromide for 2 min at room temperature and photographed by UV transillumination.

### Nested PCR-RFLP of SSU rRNA gene

For the first amplification, a reaction volume of 25 µL comprised: 2.5 µL of 10x PCR buffer (Invitrogen), 2 µL of MgCl_2_ (50 mM; Invitrogen), 0.64 µL of dNTP's mix (10 mM each; Invitrogen), 0.6 µL of each primer (40 µM), 0.2 µL (5 U/µL) of de Accu Prime™ TaqDNA Polymerase High Fidelity (Invitrogen), 2 µL (100 ng) of DNA, and 16.5 µL of sterile deionized water. Finally, 2 drops of mineral oil were added. The DNA amplification program started with 2 min of denaturation at 94°C, followed by 40 cycles of 60 s at 92°C of denaturation, primer annealing for 60 s at 47°C and extension for 90 s at 72°C; the final extension was at 72°C for 7 min. The primers used in this PCR were: Fw 5′-TTT GTA TTA GTA CAA A-3′; Rv 5′-GTA [A/G]TA TTG ATA TAC T-3′. Later, we used 4 µL of the first PCR product as a template for the nested PCR reaction with 4 µL of 10x PCR buffer (Invitrogen), 3.2 µL of MgCl_2_ (50 mM) (Invitrogen), 1 µL of dNTP's mix (10 mM each) (Invitrogen), 1 µL of each primer (40 µM), 0.2 µL (5 U/µL) of Accu Prime™ TaqDNA Polymerase High Fidelity (Invitrogen), 10.6 µL of sterile deionized water and 2 drops of mineral oil. The nested PCR was performed as described above using the first PCR conditions, except for the annealing temperature, which changed to 62°C and others primers were used: Selective for *E. histolytica* (Fw 5′-TTT AGA AAC AAT GCT TCT CT-3′ and Rv 5′- AAT GGC CAA TTC ATT CAA TG-3′) and selective for *E. dispar* (Fw 5′-AGT GGC CAA TTT ATG TAA GT-3′ and Rv 5′-TTT AGA AAC AAT GTT TCT TC-3′). Both amplifications were done in triplicate. The amplified products were stained with ethidium bromide after electrophoresis on a 1.6 % agarose gel. Positive and negative control reactions were included with each batch of samples analyzed by nested PCR. The nested PCR products of both *E. histolytica* and *E. dispar* showed approximately 874 bp fragments which correspond to small ribosomal RNA subunit (*SSU rRNA*) gene. These products were digested with the restriction endonuclease *Dra*I or *Sau*96I (5 U/µL; BIOLabs, New England) during 16 h at 37°C according to the manufacturer's instructions. The RFLP-digested product was visualized by loading 5 µL of sample on a 1.6 % agarose gel containing ethidium bromide.

## Results

### Coproparasitoscopic exam of stool samples

All samples were tested for *E. histolytica and E. dispar* using the Faust coproparasitoscopic exam. Of the 62 stool samples screened, 18 were positive for *E. histolytica,* 22 were positive for either *Escherichia coli, E. nana, Giardia lamblia*, or *Ascaris lumbricoides* cysts and negative for *Entamoeba*. Finally, 22 samples were negative for any parasite. No sample was positive for *E. dispar.*

### Specificity and sensitivity of PCR methods

**Figure 2:**
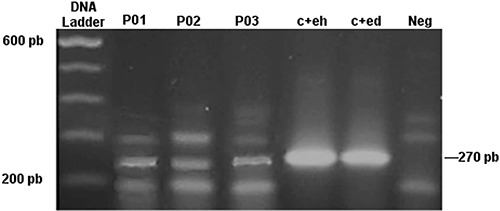
Stained agarose gel with products amplified by PCR with designed primers from samples containing *E. histolytica* or *E. dispar. Lane 1*, molecular weight marker; *lanes 2–4*, clinical samples; *lane 5*, DNA positive control for *E. histolytica* (c+eh); *lane 6*, DNA positive control for *E. dispar* (c+ed); and *lane 7*, negative control (human DNA from whole blood).

We found that the designed primers were specific for the expected fragment of 268 pb. This amplicon was observed in stool samples and positive controls (*E. histolytica* and *E. dispar*). Figure 2 shows a visible amplicon of 268pb in the analyzed samples. Although other amplicons were detected in the stool samples and negative controls, it should be noted that they were not found in the positive controls.

### Clinical evaluation of PCR-DGGE-adh112 assay

**Figure 3:**
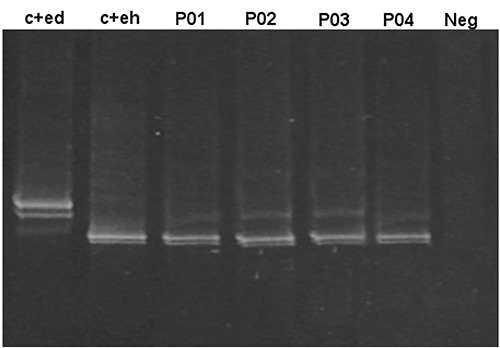
Denaturing gradient gel electrophoresis analysis of stool samples for the identification of *E*. *histolytica* and *E. dispar*. *Lane 1*, positive control of *E. dispar; lane 2*, positive control of *E. histolytica; lanes 3–6*, clinical samples; *lane 7*, negative control.

Once the DNA was isolated from stool samples, the primers were selected and concentrations standardized, the denaturant conditions were fully optimized, then DGGE gels were run (Figure 3). Out of the 62 samples, 10 (16.1%) were positive for *E. histolytica,* while 52 (83.9%) were negative. No sample was found to be positive for *E. dispar*.

### Nested PCR-RFLP of *SSU rRNA* gene

**Figure 4:**
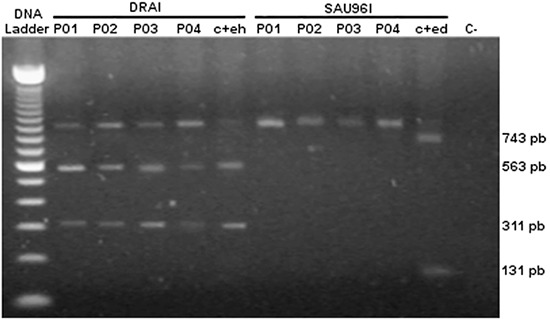
Nested PCR-RFLP (restriction fragment length polymorphism) of *SSU rRNA* gene for the identification of *E*. *histolytica* and *E. dispar*. *Lane 1*, molecular weight marker; *lanes 2–5*, *Dra*I digested PCR products; *lane 6*, DNA positive control for *E. histolytica* (c+eh); *lane 7–10*, *Sau*96I digested PCR products; *lane 11*, DNA positive control for *E. dispar* (c+ed); and *lane 12*, negative control.

The RFLP pattern for *E. histolytica* showed 563 bp and 311 bp fragments and an undigested 874 bp fragment, whereas that for *E. dispar* showed 743 bp and 131 bp fragments (Figure 4). The nested PCR-RFLP was positive for *E. histolytica* in 10 (16.1%) stool samples and 52 (83.9%) samples were negative. These results are similar to what we observed with the PCR-DGGE technique, that is, the positive samples for *E. histolytica* by PCR-DGGE were also positive by PCR-RFLP. No sample was found to be positive for E. *dispar.* The sensitivity and specificity of both, PCR-DGGE and PCR-RFLP were 100%. The 95%CI values were also estimated and used to evaluate the sensitivity (95%CI=65.55–99.08) and specificity (95%CI=91.43–99.82) of PCR-DGGE technique.

## Discussion

The identification of *Entamoeba* spp. has always been controversial and microscopy is usually used to diagnose protozoa in stool samples. However, this method is unable to differentiate *E. histolytica* from the morphologically identical non-pathogenic species such as *E. dispar* (8,14). Therefore, the WHO recommends the development and application of new methods for a specific diagnosis of *E. histolytica* infection (2). The present study describes a new PCR-DGGE strategy for species-specific detection and differentiation of *E. histolytica* and *E. dispar* DNA using stool samples. Several methods including isoenzyme analysis, antibody or antigen detection tests, immunochromatographic assays and real-time PCR have been used for an accurate detection of *E. histolytica* and *E. dispar* However, high cost limits their use in underdeveloped countries and, additionally, some yield false negative results (25–28).

Another alternative for differentiating the species is using PCR. This technique demonstrates exceptional sensitivity and specificity compared with microscopy (8,14). This research is the first of its kind in Mexico, and was designed to detect and differentiate *E. histolytica* from *E. dispar* using a fragment of the *adh112* gene, which presents five differences in single bases when comparing both species. The advantage of the PCR-DGGE-*adh112* method over the PCR and restriction enzyme digestion method has to do with the reliability of the results. The electrophoretic pattern obtained in PCR and restriction enzyme digestion are based on the nucleotides size, and it can be difficult to see the differences and similarities that could exist among numerous samples (29). In contrast, DGGE is sensitive enough to detect differences of a single base. With the PCR-DGGE-*adh112* the DNA fragments move through polyacrylamide gels containing a linear gradient of denaturing agents, resulting in a partially denatured molecule observed in the gel with distinctive electrophoretic pattern (the change in the sequence causes a change in the pattern of run), which gives greater effectiveness to the test and no likelihood of false positives or false negatives (17,24).

In Mexico, there is a lack of knowledge about the epidemiology of *E. histolytica* infection, although amebiasis has been considered for many years as a major health problem in the country (4,30). By using PCR-DGGE-*adh112* we found the mono infection with E. *histolytica* to be 16.1%. No sample was found to be positive for *E. dispar.* These results were validated using another technique (nested PCR-RFLP of *SSU rRNA* gene) considered by several authors as the gold standard for the differential diagnosis between *E. histolytica* and *E. dispar* (31–34). Our results were consistent with those observed by PCR-RFLP. Our PCR-DGGE-*adh112* results showed sensitivity and specificity of 100%, indicating that it is a useful and reliable test to specifically detect *E. histolytica* in stool samples. PCR-DGGE has the advantage of identifying and differentiating *E. histolytica* and *E. dispar*, which is not possible using microscopy or ELISA (8,35). It should be noted that the differentiation of pathogenic *E. histolytica* from the morphologically identical *E. dispar* is important for the clinical management of patients.

In conclusion, the present study reports a new PCR-DGGE technique for species-specific detection and differentiation of *E. histolytica* and *E. dispar* DNA in stool samples. This technique could become an alternative or a complementary diagnostic tool.
